# The Austin District Medical Society

**Published:** 1903-07

**Authors:** 


### Society Notes.

### The Austin District Medical Society.

The Austin District Medical Society held its regular summer meeting in Austin June 26th, ult. There was a very slim attendance, especially of the Austin members, which is a shame. Dr. Joe Wooten read an interesting paper on "Tenotomy for Club-foot," and incidentally gave Lorenz (and the "lesser lights" [?]) a rap. It provoked a lively discussion. The paper will appear in our next issue. Dr. O'Barr, of Ledbetter, read a paper on an obscure pathological condition of a patient of his, and asked for a diagnosis. A committee was appointed to get up a program for the September meeting and to stir up the doctors to attend.

